# Correction: Huang et al. Long Non-Coding RNA *74687* Regulates Meiotic Progression and Gonadal Development in Rainbow Trout (*Oncorhynchus mykiss*) via the miR-15a-5p–*ccne1* Regulatory Axis. *Int. J. Mol. Sci.* 2025, *26*, 8036

**DOI:** 10.3390/ijms27073321

**Published:** 2026-04-07

**Authors:** Tianqing Huang, Baorui Cao, Enhui Liu, Wei Gu, Yunchao Sun, Kaibo Ge, Gaochao Wang, Datian Li, Peng Fan, Ruiyan Xing, Gefeng Xu

**Affiliations:** 1 State Key Laboratory of Mariculture Biobreeding and Sustainable Goods, Heilongjiang River Fisheries Research Institute, Chinese Academy of Fishery Sciences, Harbin 150070, China; huangtianqing@hrfri.ac.cn (T.H.); c3012304124@163.com (B.C.); liuenhui@hrfri.ac.cn (E.L.); guwei@hrfri.ac.cn (W.G.); sunyunchao@hrfri.ac.cn (Y.S.); gekaibo@hrfri.ac.cn (K.G.); gaochaowang@ymail.com (G.W.); lidatian@hrfri.ac.cn (D.L.); fanpeng@hrfri.ac.cn (P.F.); 13059088876@163.com (R.X.); 2 College of Animal Science and Technology, Northeast Agricultural University, Harbin 150030, China

**Error in Figure**

In the original publication [1], there were mistakes in Figures 5 and 6 as published. In Figure 5A, the DAPI images in the fifth and sixth rows are duplicates. In Figure 6, a Western blot image from a previous paper was mistakenly inserted into Figure 6D. The corrected Figure 5 and Figure 6 appear below. The original images will be updated in the original publication as Supplementary Material. The authors state that the scientific conclusions are unaffected. This correction was approved by the Academic Editor. The original publication has also been updated.

**Insert New Supplementary Material Statement**

**Supplementary Material:** The following supporting information can be downloaded at https://www.mdpi.com/article/10.3390/ijms26168036/s1.

And the citation of Supplementary Material has been added in Sections 2.5 and 2.6 of the original manuscript.

## Figures and Tables

**Figure 5:**
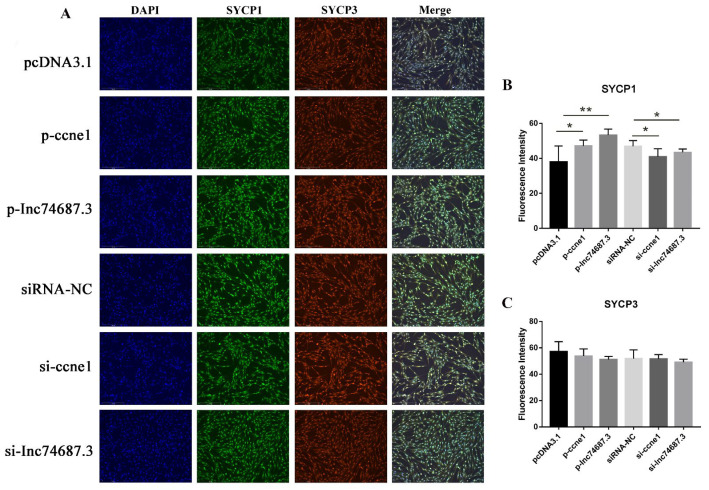
Cellular immunofluorescence detection of CCNE1, and *lnc74687.3*’s effects on expression of Synaptonemal Complex Protein 1 (SYCP1) and Synaptonemal Complex Protein 3 (SYCP3). (**A**) Immunofluorescence images of SYCP1 and SYCP3 in RTG-2 cells under different transfection conditions. (**B**) Quantification of SYCP1 fluorescence intensity by ImageJ. (**C**) Quantification of SYCP3 fluorescence intensity by ImageJ. Data are expressed as mean ± SD (*n* = 3), with differences indicated by asterisks (** *p* < 0.01, * *p* < 0.05).

**Figure 6:**
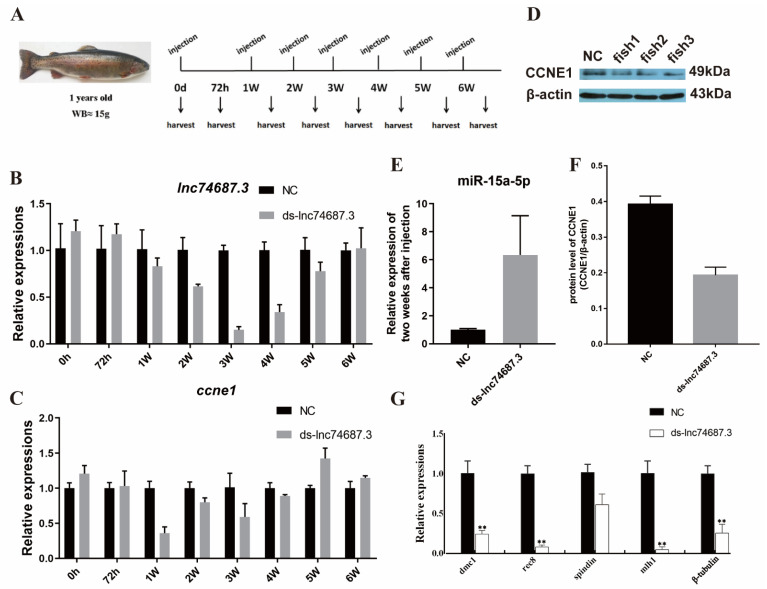
*lnc74687.3* targeting miR-15a-5p at the organismal level affects *ccne1* and CCNE1 expression. (**A**) Injection and sampling scheme; (**B**) *lnc74687.3* expression in rainbow trout gonads at different time points; (**C**) *ccne1* expression in rainbow trout gonads at different time points; (**D**) CCNE1 protein expression levels at weeks 1, 2, and 5; (**E**) miR-15a-5p expression two weeks after *lnc74687.3* knockdown; (**F**) CCNE1 protein expression levels; (**G**) effect of *lnc74687.3* knockdown on meiosis-related genes *dmc1*, *rec8*, *spindlin*, *β-tubulin*, and *mlh1*. Data are expressed as mean ± SD (*n* = 3), with differences indicated by asterisks (** *p* < 0.01).
